# Calreticulin Identified as One of the Androgen Response Genes That Trigger Full Regeneration of the Only Capable Mammalian Organ, the Deer Antler

**DOI:** 10.3389/fcell.2022.862841

**Published:** 2022-06-13

**Authors:** Qianqian Guo, Junjun Zheng, Hengxing Ba, Hongmei Sun, Jingjie Zhai, Wenying Wang, Chunyi Li

**Affiliations:** ^1^ Institute of Antler Science and Product Technology, Changchun Sci-Tech University, Jilin, China; ^2^ Institute of Special Economic Animal and Plant Sciences, Chinese Academy of Agricultural Sciences, Jilin, China; ^3^ Department of Oral Implantology, Jilin Provincial Key Laboratory of Sciences and Technology for Stomatology Nanoengineering, Hospital of Stomatology, Jilin University, Jilin, China

**Keywords:** antler regeneration, calreticulin, androgen response gene, antler stem cells, RNAi

## Abstract

Deer antlers are male secondary sexual characters that develop to become bone; they are unique appendages that, once lost, can fully regenerate from the permanent bony protuberances or pedicles. Pedicle periosteum (PP) is the tissue that gives rise to the regenerating antlers with three differentiation stages, namely, dormant (DoPP), potentiated (PoPP), and activated (AcPP). Thus far, the transition from the PoPP to the AcPP has not been studied. Our results showed that the AcPP cells maintained their original stem cell features by expressing mesenchymal stem cell (MSC) markers CD73, CD90, and CD105, although they had entered the proliferation mode. The differentially expressed genes (DEGs) in the AcPP compared with those of the PoPP were mainly involved in protein processing, cell cycle, and calcium signaling pathways. Calreticulin (CALR), an androgen response gene, was significantly differentially upregulated in the AcPP cells, and its expression level was negatively regulated by androgens, in contrast to the currently known model systems where all regulation is positive. The downregulation of CALR expression in the AcPP cells *in vitro* inhibited cell proliferation, induced apoptosis, and inhibited cell cycle progression at G1-S transition. Therefore, CALR is likely a downstream mediator of androgen hormones for triggering initiation of antler regeneration. We believe that the identification of CALR has not only discovered “one critical piece” of the “jigsaw puzzle” in the initiation of antler regeneration but also helps in revealing the mechanism underlying this unique mammalian epimorphic regeneration and has also opened a new avenue for the study of the nature of CALR regulation by androgen (putative binding partners), thus facilitating the identification of potential molecule(s) for investigation as targets for clinical evaluation.

## Introduction

Deer antlers are the only mammalian organ that, once lost, can fully grow back (Goss and Rosen, 1973; Price et al., 2005). Thus, they offer a unique opportunity to explore how nature has solved the problem of mammalian organ regeneration, a matter highly pertinent to the field of regenerative medicine (Nieto-Diaz et al., 2012). Each year in spring, hard antlers are cast from the permanent bony protuberances (pedicles), which triggers the regeneration of new (velvet) antlers (Kierdorf et al., 2009; Kierdorf and Kierdorf, 2011). Total or partial deletion of the pedicle periosteum (PP) prior to antler regeneration has demonstrated convincingly that it is the PP that gives rise to regenerating antlers (Li et al., 2007). Membrane insertion experiments have shown that to enable the antler to regenerate, the PP must interact with its enveloping skin, which is facilitated by close association of these two tissue types (Li et al., 2008). Based on these findings, Li et al. (2004) and Li et al. (2007) classified the PP along the longitudinal axis of a pedicle into three states proximo-distally, the dormant PP (DoPP, proximal two-thirds of the PP), characterized by loosely linked to the pedicle skin; the potentiated PP (PoPP, distal third of the PP), intimately associated with the pedicle skin; and the activated PP (AcPP, peripherally encroaching into the cast plane of the pedicle), which is intimately associated with the antler (velvet) skin.

In seeking to unveil the molecular mechanism underlying the transition from the DoPP to the PoPP, our team has carried out a series of studies. Dong et al. (2016) identified 169 differentially expressed proteins (70 upregulated and 99 downregulated) of the PoPP over the DoPP using iTRAQ-based quantitative proteomic analysis. Yang et al. (2016) found that the levels of genome-wide DNA methylation were significantly lower in the PoPP than in the DoPP. Furthermore, Dong et al. (2019) reported that the epithelial–mesenchymal transition process may participate in potentiation of the PP (using two-dimensional difference gel electrophoresis, 2D-DIGE). All of these results are consistent with our histological (Li et al., 2005) and tissue deletion (Li et al., 2007) findings. However, thus far, the mechanism underlying the transition from the PoPP to the AcPP has not been resolved, albeit that it is the indispensable step for initiation of antler blastema formation and subsequent regeneration of the antler (Li and Chu, 2016).

Antlers and their antecedent pedicles are male secondary sexual characters and, as such, their development is strictly under control of androgen hormones (Weerasekera et al., 2020). Surprisingly, except for pedicle initiation and antler calcification that require high levels of androgens, androgens seem to be detrimental to antler regeneration and growth. In this respect, initiation of antler regeneration can only start when androgens decrease to an almost undetectable level; castration at any time of stags in the hard antler stage will induce casting of the hard antler and immediate regeneration of a new antler, and further administration of exogenous androgen can effectively suppress antler regeneration (Bubenik et al., 1982; Suttie et al., 1995; Akhtar et al., 2019). Therefore, androgens must act as a “brake” on antler regeneration likely through suppression of the activation of the PP cells. However, the identity of such androgen response gene(s) that are released from the “brake” is, thus far, unknown.

The aim of the present study was to use a range of techniques to investigate the transition from the PoPP state to the AcPP state (triggering of PP cell proliferation and thus initiation of antler regeneration) and identify candidates for the androgen response gene(s) through the analysis of differential expression. Our results showed that the AcPP cells, which had entered the proliferation state, still partly maintained the original stemness evidenced by the expression of marker genes of the mesenchymal stem cells (MSCs; CD73, CD90, and CD105). Furthermore, during this transition, calreticulin (CALR), an androgen response gene, was highly expressed in the AcPP cells. The downregulation of CALR *in vitro* had significant influence on the PP cells evidenced by reduction in the cell proliferation rate, inhibition of cell cycle progression, and induction of apoptosis. Consequently, we propose that CALR is a key androgen response factor that triggers the initiation of antler regeneration *in vivo*.

## Materials and Methods

### Ethics Statement

All animal-related experiments in the present study were performed in accordance with the guidelines of the Animal Care and Use Committee of Jilin University and under the approval from the Temporary Animal Ethics Committee of Changchun Sci-Tech University (Permit Number: CKARI-2020-017).

### Experimental Animals and Tissue Sampling

The 3-year-old sika deer (*Cervus nippon*) stags were used in the study from Dong Ao Deer Farm. Three stags were selected from the herd at the time of hard antler button casting when the distal rim of the pedicle skin had encroached onto the casting surface peripherally and occupied one-third to one-half of the entire casting surface (Supplementary Figure S1A). Each selected animal was slaughtered immediately; the right-side pedicles were used for histology and cell culture and the left-side ones for molecular study. The detailed procedures for sampling of PoPP and AcPP tissues were reported elsewhere (Li et al., 2003). The tissue samples from the right-side pedicles were fixed in 10% formalin for histology; those from the left side were frozen in liquid nitrogen and stored at −80°C for RNA extraction and sequencing.

### Histology

Histological procedures for processing PP tissues have been reported elsewhere (Li et al., 2005). In brief, trimmed PP tissues were embedded in paraffin wax, sectioned at 4 μm, rehydrated, and co-stained with Alcian blue (AB) and hematoxylin/eosin (HE). The stained sections were then dehydrated, mounted, examined, and photographed under a slice scanner (Leica, Germany).

### Immunohistochemistry

IHC was performed using the UltraSensitive TM SP (Mouse/Rabbit) IHC Kit (MX Biotechnologies, China), according to the manufacturer’s protocol. Briefly, PP tissue sections were rehydrated and subjected to antigen retrieval. After blocking the nonspecific binding with milk powder, the sections were incubated with primary antibodies overnight at 4°C and washed with PBS three times; the sections were then incubated with biotinylated secondary antibody (MX Biotechnologies, China) for 10 min at room temperature and finally visualized using DAB (3, 3–diaminobenzidine; MX Biotechnologies, China) staining. First antibodies used in this study included rabbit anti-ki67 (1:1000, Abcam, ab16667), rabbit anti-CD90 (1:100, Bioss, bs-0778R), mouse anti-CD105 (1:100, Elabscience, ESH135), rabbit anti-CD73 (1:100, Santa, sc-25603), and rabbit anticalreticulin (CALR; 1:50, Abcam, ab702); isotypic IgG (1:500, Abcam, ab172730) was used as the control. The images were captured by using an inverted microscope (Nikon, Japan) or a slice scanner (Leica, Germany).

### RNA-Seq

RNA-sequencing (RNA-seq) was performed by BGI Life Tech Co. Ltd. (Wuhan, China). The PP tissues were rapidly ground into fine powder in liquid nitrogen using Freezer/Mill 6770 (SPEX CertiPrep Ltd., United States), and total RNA was extracted using TRIzol reagent (Qiagen, Hilden, Germany) according to the manufacturer’s procedure. RNA quality was confirmed using a bioanalyzer with a minimum RNA integrity number of 7. In total, 0.6 mg RNA was used to construct libraries according to the manufacturer’s instructions (Illumina TruSeq Library Preparation Kit v3), and libraries were sequenced using an Illumina HiSeq X Ten at BGI (Shenzhen, China). SOAPnuke (v1.5.2; Cock et al., 2009) was used to filter raw reads, and high-quality reads were aligned to the *Cervus hanglu yarkandensis* (Yarkand deer) reference genome CEY_v1 (Ba et al., 2020) by HISAT (v2.0.4; Kim et al., 2015). The transcript quantification (FPKM, fragments per kilobase per million) was calculated using RSEM (Li and Dewey, 2011). Analysis of the differential expression gene (DEG) was performed using DEGseq2 software (v1.4.5; Love et al., 2014). Genes meeting the criteria set at |log2 (fold change)|>0.5, and the false discovery rate (FDR) < 0.01 were assigned as differentially expressed. Principal components analysis (PCA) was performed with the R packages factoextra v1.0.6 and FactoMineR v2.2. Venn diagram and the volcano plot were drawn using Dr. Tom software (https://biosys.bgi.com). GO and KEGG enrichment analyses were carried out using the R package clusterProfiler (v3.4.4; Yu et al., 2012).

### Cell Culture

Procedures for culturing the AcPP and PoPP cells were as described previously (Li et al., 2012; Seo et al., 2014; Guo et al., 2015). Briefly, the cells were cultured in the medium containing DMEM (Life, United States) plus 10% FBS (Gibco, United States), 100 U/ml penicillin, and 100 μg/ml streptomycin (Invitrogen, United States) at 37°C in 5% CO_2_, passaged using trypsin (Sigma, United States) and stored in liquid nitrogen in freezing medium (90% FBS +10% DMSO). When required, the cells were thawed and seeded in T75 flasks (Nest Biotechnology, United States). The cells used in this study had gone through two passages.

### Testosterone Treatment, Cell Proliferation, and Calreticulin Expression Assays

The cells were seeded in 24-well plates at a density of 2 × 10^4^ cells/ml/well. Each treatment was performed in triplicate. The cells were incubated for 48 h and followed by 24 h starvation in the serum-free medium (culture medium without FBS) prior to the following treatments for 24 h: 1) 1% FBS, 2) 5% FBS, 3) 10% FBS, 4) 0.0 nM T, 5) 0.5 nM T (Sigma, United States), 6) 1.0 nM T, 7) 5.0 nM T, 8) 10.0 nM T, and 9) 50.0 nM T. The cell proliferation rate and the expression level of CALR were analyzed by CCK-8 and qRT-PCR assays, respectively.

### Cell Transfection

Three short-hairpin RNAs (shRNA) targeting CALR (shCALR1: GCG​GCC​TGA​TAA​TAC​CTA​T, shCALR2: GCT​GGA​TCG​AAT​CCA​AAC​A, and shCALR3: GCT​GGA​TCG​AAT​CCA​AAC​A) were synthesized by GenePharma Biotechnology (Shanghai, China). The 293 T-cells were transiently transfected with the recombinant lentiviral shRNA constructs or empty carrier (pLVTHM as negative control) along with the pCMV∆8.9 and pMD2G plasmids in a ratio of 2:2:1 using Lipofectamine 3000 (Invitrogen, United States) according to the manufacturer’s protocol. Virus-containing supernatants were collected at 24 and 48 h after transfection, pooled together, and then concentrated by centrifugation (5,000 g, 40 min) using the Amicon ultra centrifugal filter devices (Millipore Corporation, United States). Then, the AcPP cells were infected with the lentiviruses carrying shCALR1, shCALR2, shCALR3, or the negative control in the presence of 5 μg/ml polybrene (Sigma-Aldrich). The GFP expressing cells were sorted by flow cytometry (BD FACSAria, United States) according to the manufacturer’s manual. The efficiency of CALR silencing by these shRNAs was determined using Western blotting and qRT-PCR assays.

### Western Blot Analysis

Total cellular proteins were extracted using RIPA buffer, and the cell lysates were centrifuged at 15,000 g at 4°C for 15 min. The protein concentration was measured using the BCA protein assay (Beyotime, China). Then, 30 µg protein was separated on a 10% SDS-PAGE, and the separated proteins were electro-transferred onto the 0.2-µm PVDF membranes. The membranes were then blocked in TBST containing 5% nonfat dry milk for 2 h at room temperature and hybridized with the primary antibody (rabbit anti-CALR, Abcam) overnight at 4°C. Then, the blots were probed with HRP-conjugated secondary antibodies and detected using ECL, photographed, and quantified using ImageJ software.

### Quantitative Real-Time PCR

Total RNA was isolated using TRizol reagent and purified on a silica base spin column (SK1321, Shanghai Shenggong Inc., China) according to the manufacturer’s protocol. The specific primers, based on the DNA sequences located in the gene coding regions, were designed using software Primer 5 (Supplementary Table S1). Glyceraldehyde-3-phosphate dehydrogenase (GAPDH) was used as an endogenous control. Total RNA was reverse-transcribed onto cDNA using the cDNA Synthesis Kit (Invitrogen Inc., Camarillo, CA, United States). The SYBR Kit (Applied Biosystems, Foster City, CA, United States) was used in the qRT-PCR assay according to the manufacturer’s protocol. Relative expression was calculated using the 2^−ΔΔCT^ method to assess the fold change in expression levels of the target genes. Linear regression analysis was performed using the ggplot2 R package (Bader and Hogue, 2003).

### Cell Counting Kit-8 and Cell Colony Formation Assays

Cell viability was measured using the Cell Counting Kit-8 (Solarbio, China) according to the manufacturer’s instructions. The colony formation assay was carried out using a reported methodology (Wang et al., 2019). Briefly, 200 cells in 2 ml medium were seeded into each well of 6-well plates, and the medium was replaced every 4 days; after 14 days of culture, the formed cell colonies were washed with PBS, fixed in 4% paraformaldehyde for 30 min, and stained with 0.5% crystal violet dye for 5 min. Nonspecific staining was removed by three rinses with double-distilled water, and the cells were photographed under a microscope. The colony-forming ability of cells was estimated by quantifying the number of colony-forming units (CFUs, ≥50 cells).

### EdU (5-Ethynyl-20-Deoxyuridine) Incorporation Assay

The EdU incorporation assay was performed using a KeyFluor594 Click-iT EdU Kit (KeyGEN BioTECH, China) according to the manufacturer’s protocol. Briefly, the cells were first incubated with the medium supplemented with 50 µM EdU for 3 h and fixed in 4% polyformaldehyde for 30 min. The cells were then permeabilized with 0.5% Triton X-100 and subsequently blocked with 3% bovine serum albumin (BSA) in PBS for 2 h. The nuclei of cells were counterstained with DAPI for 5 min in the dark. The specific fluorescent staining was examined under a fluorescent microscope.

### Flow Cytometry Analysis

Flow cytometry was used to analyze the cell cycle and cell apoptosis. For cell cycle analysis, the cells were collected and fixed in pre-cooled 75% ethanol overnight at −20°C, centrifuged at 500 g, and resuspended in 500 µl PBS containing 10 µl RNase (50 μg/ml; Sigma, United States) for 30 min at 37°C, followed by the incubation with propidium iodide (PI, final concentration: 50 μg/ml) solution for 30 min in the dark, and then cell cycles were analyzed using flow cytometry.

Apoptosis was detected using the Annexin V-PE/7-AAD staining kit (Kaiji Inc., China), according to the manufacturer’s instruction. Briefly, 1–2 × 10^6^ cells were trypsinized using EDTA-free trypsin (Invitrogen, United States) and centrifuged at 1,500 g, washed twice in 10 ml PBS, and then labeled with 7-AAD and Annexin V-PE in binding buffer according to the manufacturer’s instructions. The apoptotic cells were detected using flow cytometry.

### Statistical Analysis

The results are presented as mean ± SD. Statistical significance was evaluated using GraphPad Prism 8.0.1 (GraphPad Software, La Jolla, CA, United States) software. Statistical analysis for the comparisons of multiple variables was performed using a two-way ANOVA, and Student’s *t-*test was used to compare two variables. All experiments were performed in triplicate. The values were set at *p* < 0.05 for statistical significance.

## Results

### Activated Pedicle Periosteum Cells Were in the Proliferation Mode but Retained the Expression of Classical Mesenchymal Stem Cell Marker Genes: CD73, CD90, and CD105

**FIGURE 1:**
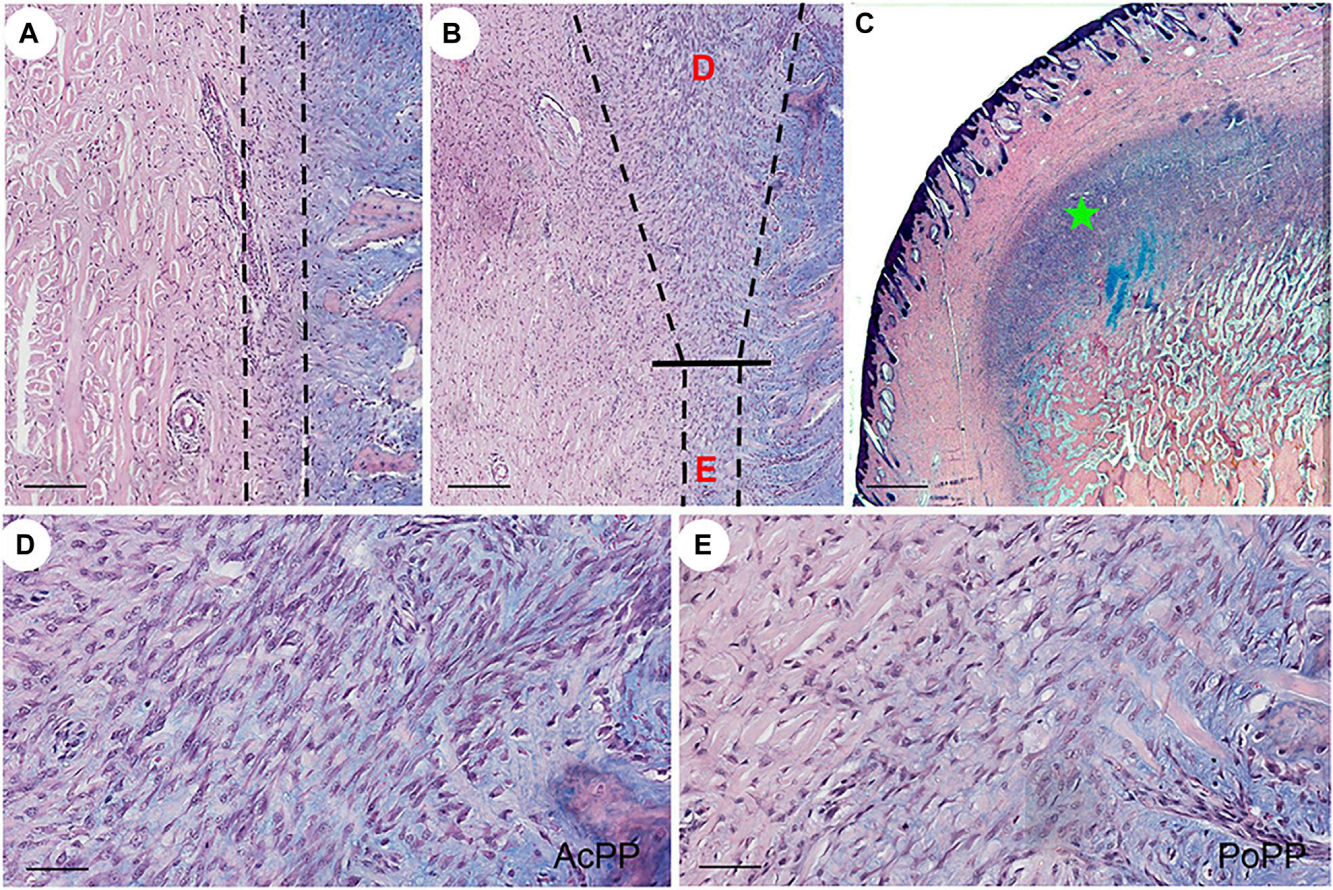
Histological portrayal of PP differentiation during the initiation of antler regeneration. Histological sections of the distal pedicle tissue after hard antler casting. **(A)** Dormant PP (DoPP) encompassed within the dotted lines. **(B)** Potentiated PP (PoPP, “E”) and activated PP (AcPP; “D”) within the dotted lines. **(C)** AcPP (“green star”). HE and Alcian blue staining. **(D,E)** Higher magnification of the areas “D” and “E” in **(B)**. It is to be noted that cells in the AcPP are much more densely populated than in the PoPP. Scale bar = 120 µm **(A,B)**, 1 mm **(C)**, and 50 µm **(D,E)**.

On the histologically processed tissue sections, three developmental states of the PP [DoPP (Figure 1A), PoPP (Figure 1B), and AcPP (Figures 1B,C)] were clearly demarcated. At a higher magnification, one could clearly see that the AcPP layer was much thickened (Figure 1B), and cells in the AcPP (Figure 1D) were more densely populated than those in the PoPP (Figure 1E). This indicates that immediately after hard antler casting, transition from the PoPP to the AcPP had occurred and antler regeneration had started.

**FIGURE 2:**
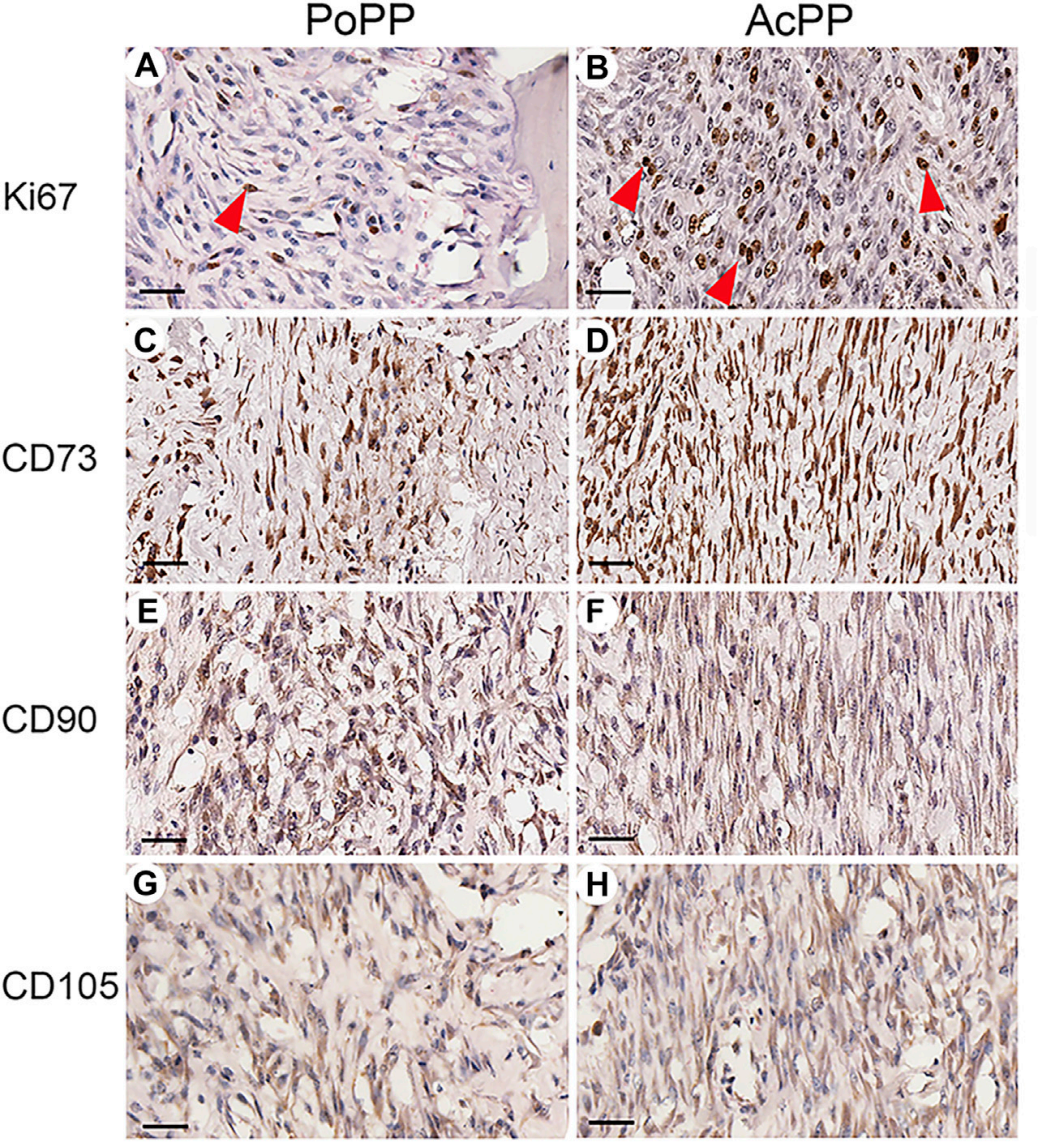
Immunohistochemistry (IHC) of Ki67 and MSC marker genes for the PoPP and AcPP. PoPP: **(A,C,E,G)**; AcPP: **(B,D,F,H)**, **(A,B)** IHC staining of Ki67 (red arrowheads). **(C,D)** IHC staining of CD73. **(E,F)** IHC staining of CD90. and **(G,H)** IHC staining of CD105. Scale bar = 50 µm.

To further confirm the histological results (i.e., that AcPP cells were in proliferation mode), we carried out IHC staining of Ki67, with a highly significant difference in the mitotic figures being detected; this was higher in the AcPP than in the PoPP (Figures 2A,B). To determine whether the AcPP cells (proliferation mode) retained their original stemness as per their antecedents, the PoPP cells, we examined the expression of three classic MSC marker genes: CD73, CD90, and CD105 (Wang et al., 2019). The results showed that all three MSC marker genes were positively stained in almost all cells of the AcPP, with no visible difference in expression levels between the AcPP and PoPP cells (Figures 2C–H). Therefore, even after entering the proliferation mode, PP cells still retained their MSC features with the expression of classic MSC marker genes at the AcPP stage.

### Significant Number of Differentially Expressed Genes Identified in the Cells of the Activated Pedicle Periosteum Over the Potentiated Pedicle Periosteum

**FIGURE 3:**
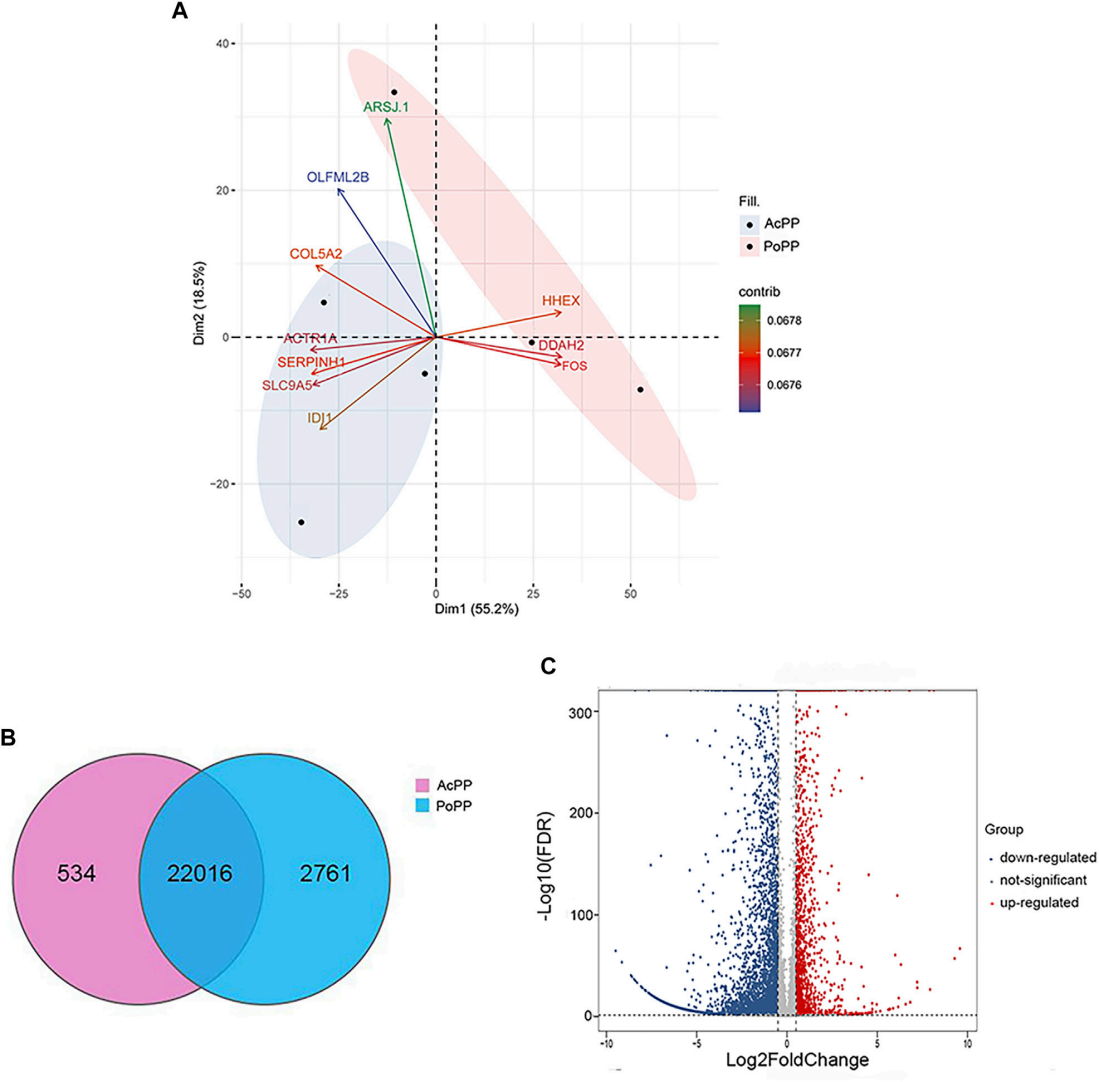
Analyses of the differentially expressed genes (DEGs) in the AcPP compared with those in the PoPP. **(A)** Principal component analysis (PCA) of DEGs in the six PP samples. The first two eigenvalues were plotted with data ellipses for each sample using the confidence intervals of 0.95. It is to be noted that the PCA results clearly separated the AcPP group (blue ellipse) from the PoPP group (pink ellipse); and the biplot showed the top ten genes contributing to the PCA in Panel. **(B)** Venn diagram showing the total number of expressed genes in the AcPP and the PoPP. The overlapping region indicates the shared genes (22,016); 534 genes were specifically expressed in the AcPP and 2,761 genes in the PoPP. **(C)** Volcano plot showing downregulated (blue) and upregulated (red) DEGs based on the criteria set at |log2 (fold change)|>0.5 and FDR < 0.01 as cutoff.

A total of 42.1 Gbps of clean paired-end reads from six libraries (triplicates/group) were obtained after quality filtering (Supplementary Table S2). These clean reads were aligned to the reference genome, with an alignment rate of 88.66 ± 2.10% (Supplementary Table S3). Principal component analysis (PCA) was carried out to determine the spatial distribution and similarities between the AcPP and PoPP. We found that different samples of the same tissue type were unambiguously grouped together (Figure 3A). In total, 25,311 genes were detected; of these genes, 22,016 were co-expressed in both the AcPP and the PoPP and 534 and 2,761 genes were specifically expressed in the AcPP and in the PoPP, respectively (Figure 3B). In total, 6,214 DEGs between the AcPP and PoPP were identified through RNA-seq, including 1,548 upregulated and 4,666 downregulated based on the criteria set at |log2 (fold change)|> 0.5 and FDR < 0.01. The distribution status of the identified DEGs was visualized using a volcano plot (Figure 3C).

### Predicted Functions of the Differentially Expressed Genes in the Activated Pedicle Periosteum Through Analyses of Gene Ontology and Kyoto Encyclopedia of Genes and Genomes

**FIGURE 4:**
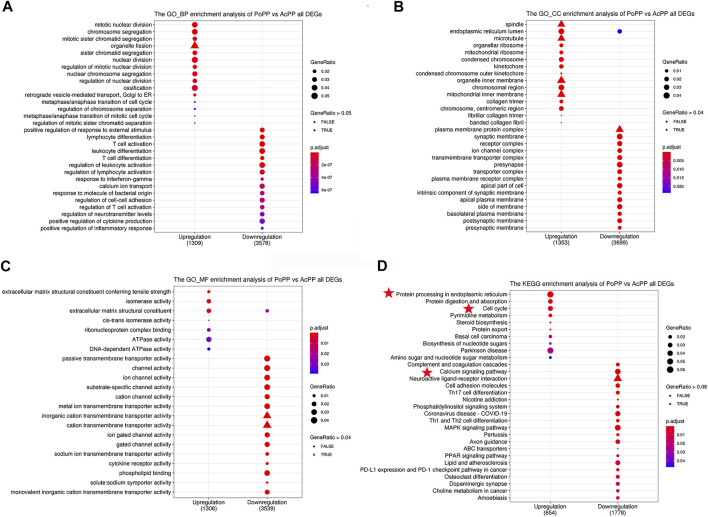
GO and KEGG pathway analyses of all DEGs in the AcPP and the PoPP. **(A–C)** GO enrichment analysis: **(A)** biological process (BP), **(B)** cellular composition (CC), and **(C)** molecular function (MF). **(D)** KEGG enrichment analysis: it is to be noted that the pathways marked by red stars may play important roles in the activation from the PoPP to the AcPP. AcPP: activated pedicle periosteum; PoPP: potentiated pedicle periosteum.

To predict the functions of the identified DEGs in the AcPP, both Gene Ontology (GO) and Kyoto Encyclopedia of Genes and Genomes (KEGG) enrichment analyses were performed. The top significantly upregulated BP terms were mainly related to the activation of cell proliferation and osteogenesis: mitotic nuclear division, chromosome segregation, organelle fission, mitotic sister chromatid segregation, sister chromatid segregation, and ossification (Figure 4A). These findings are consistent with the results of our histological observations, in that AcPP layers were significantly thickened and the number of proliferating cells was significantly increased. In contrast, the significantly downregulated BP terms were mainly related to the inhibition of immune response: T-cell activation, regulation of leukocyte activation, regulation of lymphocyte activation, and positive regulation of inflammatory response. In addition, DEGs involved in CC terms showed significant enrichment in the endoplasmic reticulum lumen, ribosome, and plasma membrane (Figure 4B), and DEGs involved in MF terms were mainly related to extracellular matrix structural constituent, channel activity, and cytokine receptor activity (Figure 4C).

KEGG analysis results showed that the most significant pathways for the upregulated DEGs were “protein procession in the endoplasmic reticulum” (e.g., CALR), “cell cycle” (e.g., CDK1, CCNB1, and E2F1), and “protein digestion and absorption” (e.g., SLC1A5; Figure 4D), which were consistent with the results of GO analysis, in that the upregulated DEGs were enriched in the biological process related to “cell division.” The downregulated DEGs were mainly enriched in the pathways including “the calcium signaling pathway” (e.g., IP3R, CAMK, and PKC) and “Th1 and Th2 cell differentiation” (e.g., IL6 and CXCL12).

### Validation of RNA-Seq Results

These RNA-seq results were validated using qRT-PCR, and the androgen response gene CALR was identified as one of the highly expressed DEGs in the AcPP.

**FIGURE 5:**
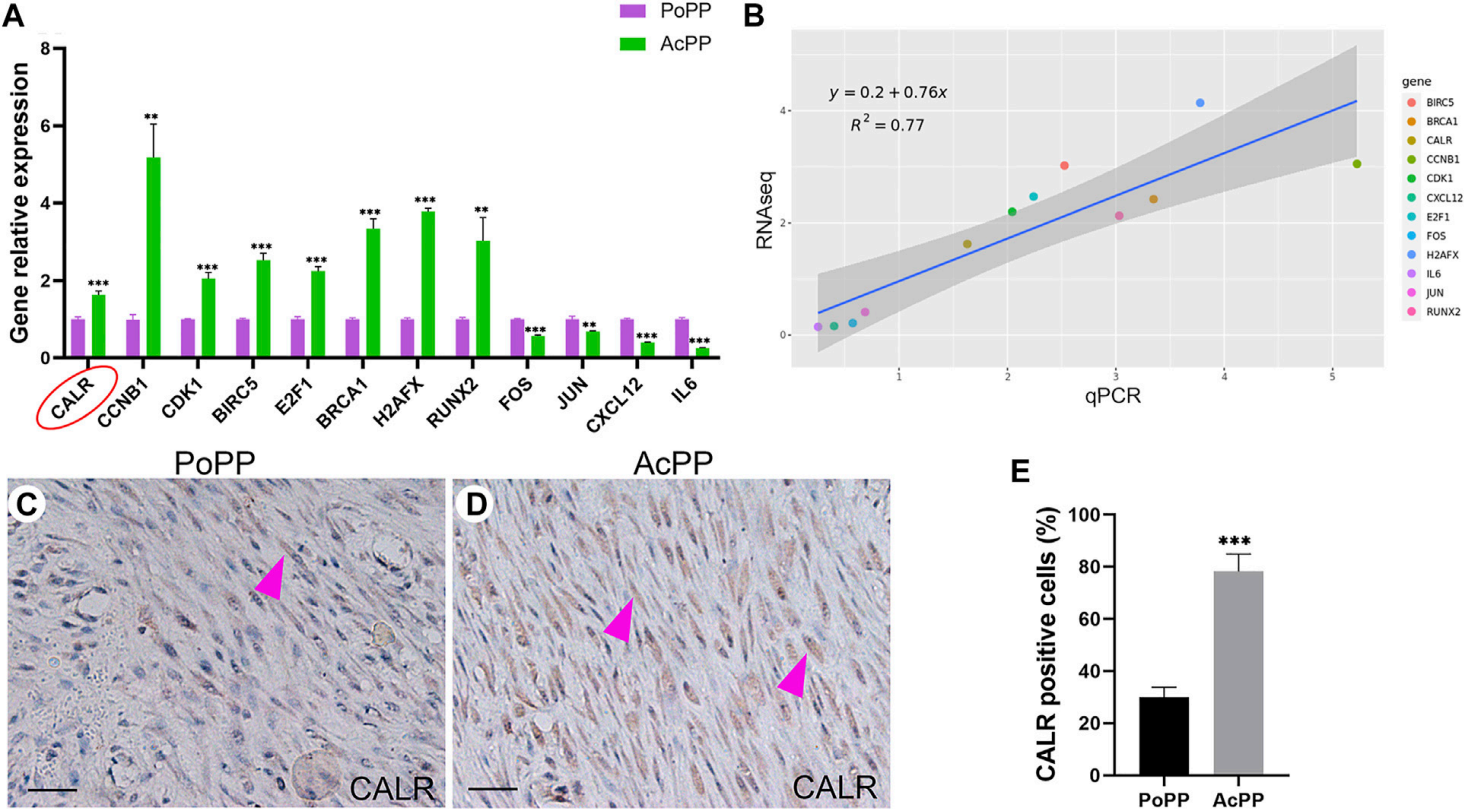
RNA-seq results validated using qRT-PCR; CALR was highly significantly upregulated in the AcPP compared with the PoPP. **(A)** Histogram showing the mRNA expression levels of 12 DEGs (CCNB1, CDK1, E2F, RUNX2, BRCA1, H2AFX, CALR, FOS, JUN, BIRC5, IL6, and CXCL12, relative to that of GAPDH) in the AcPP (green) and the PoPP (pink) using qPCR. **: *p* < 0.01 and ***: *p* < 0.001. **(B)** Correlation analyses between the RNA-seq and qRT-PCR for the 12 DEGs, where a high correlation was detected (*R* ^*2*^ = 0.77). **(C,D)** IHC of calreticulin (CALR); it is to be noted that weak positive staining was observed in cells that are sparsely populated in the PoPP but strong positive staining in cells densely populated in the AcPP. Scale bar = 50 µm. **(E)** Percentage of CALR positive cells; it is to be noted that the percentage of CALR positive cells in the AcPP was significantly higher than that in the PoPP. ***p < 0.001.

Twelve key DEGs involved in the enriched signaling pathways or biological processes were selected for the verification through qRT-PCR. The results showed that expression levels of these selected DEGs from RNA-seq were confirmed being highly consistent (*R* ^2^ = 0.77) with those from qRT-PCR (Figures 5A,B), indicating the reliability of our RNA-seq data. Among the verified DEGs, CALR was found to be one of the androgen-responsive genes and was highly differentially expressed in the AcPP cells over the PoPP cells (Figure 5A), being involved in both the upregulated pathway “protein procession in the endoplasmic reticulum” and the downregulated pathway “calcium signaling pathway” (Figure 4D). Given that antlers are the male secondary sexual characters and CALR is the androgen responsive gene, this gene was, therefore, selected for further analysis.

To further confirm the findings for CALR from both RNA-seq and qRT-PCR, we carried out IHC staining. The results showed that CALR-positively stained cells were only sparsely scattered in the PoPP (Figure 5C) whereas densely populated in the AcPP (Figure 5D). Cell counting results (Figure 5E) showed that the percentage of CALR-positive cells in the AcPP (78.22% ± 6.68%) was significantly more (*p* < 0.001) than that in the PoPP (30.00% ± 3.82%). Taken together, these results indicate that CALR is significantly upregulated during the activation process from the PoPP to the AcPP and may play a key role in the initiation of antler regeneration *via* the promotion of PP cell proliferation.

### Expression of Calreticulin Was not Only Associated With Changes in Androgen Hormones but Also Causally Related to Them

**FIGURE 6:**
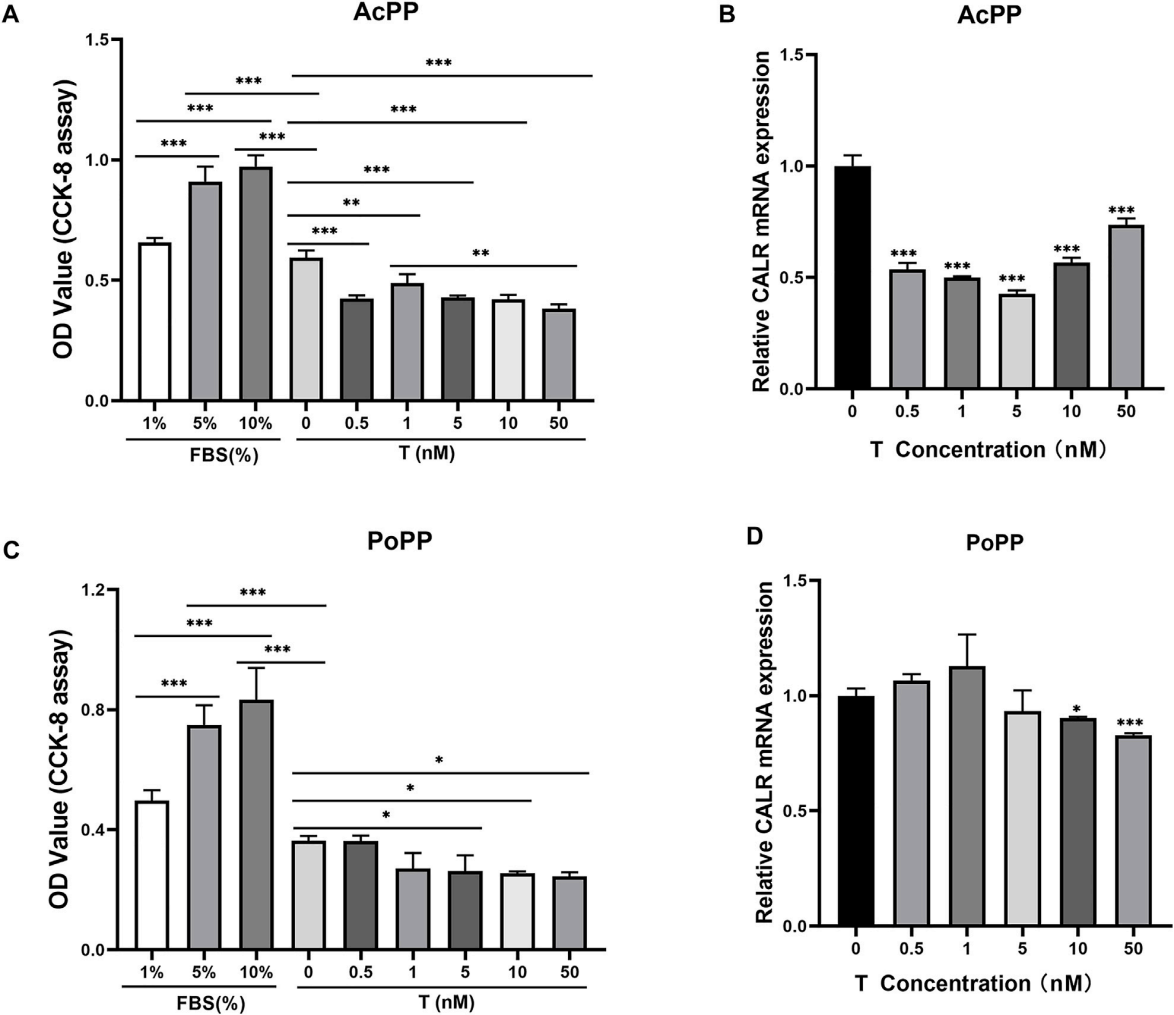
Effects of testosterone (T) treatment on cell proliferation and CALR mRNA expression in the cells of the AcPP and the PoPP. **(A,C)** Effects of T (0.5–50 nM) on cell proliferation of the AcPP **(A)** and the PoPP **(C)**, respectively. Expression profiles of CALR mRNA in the AcPP **(B)** and PoPP **(D)** cells in serum-free medium with different concentrations (0.5–50 nM) of T; it is to be noted that T treatment decreased levels of CALR expression in these PP cells, with the effect being much greater in the AcPP than in the PoPP. *p < 0.05, **p < 0.01, and ***p < 0.001.

To determine whether upregulation of CALR in the AcPP is causally related to a decreasing level of circulating androgen hormones during the period of initiation of antler regeneration, we carried out an *in vitro* assay. The results showed that the addition of a wide range of concentrations (0.5–50 nM) of testosterone (T) in serum-free medium, including physiological concentration, significantly suppressed the proliferation of both the AcPP (*p* < 0.01; Figure 6A) and PoPP (*p* < 0.05; Figure 6C) cells compared with those of the control. This shows that the high level of T negatively impacts PP cell growth, hence inhibiting the initiation of antler regeneration. This result is consistent with the *in vivo* findings that antler regeneration always takes place when circulating T reaches almost an undetectable level, and administration of exogenous T at the antler growth season can effectively prevent the activation of antler regeneration (Bubenik et al., 1982; Suttie et al., 1995). Notably, in the present study, a high level of T effectively downregulated the expression of CALR in the AcPP (*p* < 0.001; Figure 6B) and the PoPP (*p* < 0.05; Figure 6D) compared to that in the control. Our results further confirmed that CALR is the androgen-responsive gene in the antler system and is negatively regulated by androgens.

### Downregulation of Calreticulin Expression Significantly Impaired Proliferation of the Activated Pedicle Periosteum Cells

**FIGURE 7:**
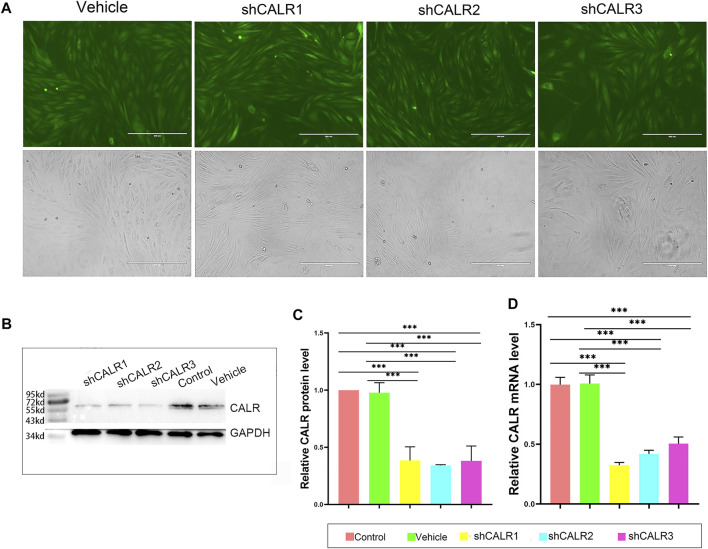
Effects of RNAi on the CALR expression in the AcPP cells. **(A)** GFP expression profiles 48 h after lentiviral infection; it is to be noted that fluorescence was clearly observable in both shRNA-CALR infected and empty vector-infected (vehicle) cells; Scale bars = 400 µm. **(B)** Expression profiles of CALR protein in the AcPP cells after transfection with shCALR1, shCALR2, shCALR3, or empty vector (vehicle) using Western blot analysis. **(C)** Relative expression levels of CALR protein in the AcPP cells; it is to be noted that shCALR1, shCALR2, or shCALR3 significantly downregulated CALR expression. **(D)** Relative expression levels of CALR mRNA; it is to be noted that the results are consistent with those of Western blot analysis, and the most effective one is shCALR1. *p < 0.05 and ***p < 0.001.

To test the direct effects of CALR on PP cell proliferation and initiation of antler regeneration, the expression of CALR in the AcPP cells was downregulated through RNAi. The results showed that all recombinant lentiviral transfections were successful (fluorescence; Figure 7A); all three constructed target sequences (shCALR1, shCALR2, and shCALR3) significantly downregulated the expression of CALR at both protein (*p* < 0.01; Figures 7B,C) and mRNA (*p* < 0.001; Figure 7D) levels in the AcPP cells compared to the empty vector shRNA (vehicle) and untreated (control) groups, and shCALR1 was found to be the most effective (knockdown rate: 67.50 ± 2.25%) and therefore was selected for further study and denoted as the CALR-RNAi group. No significant difference was detected between the vehicle and control groups in the knockdown rate (*p* > 0.05).

**FIGURE 8:**
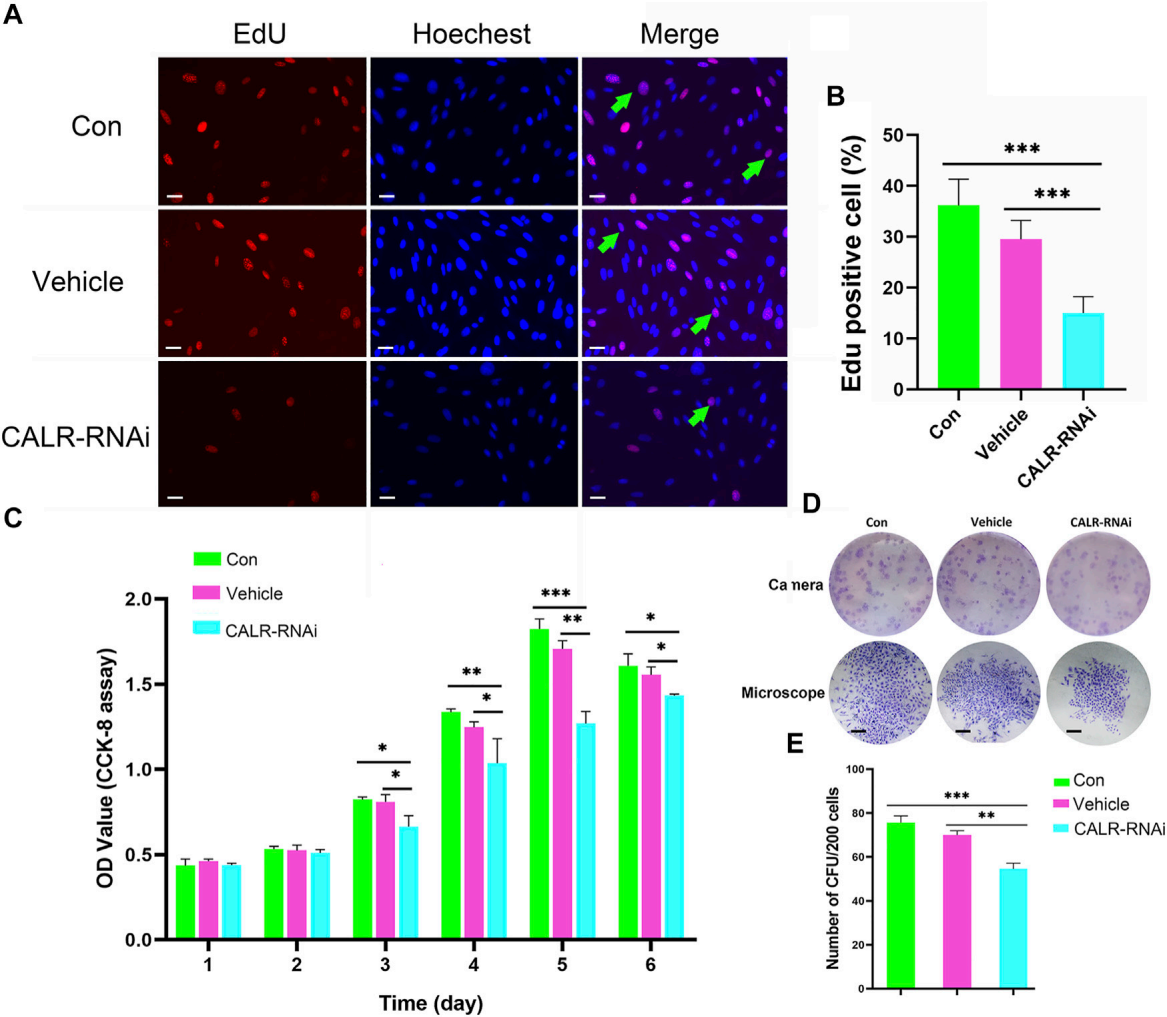
Effects of CALR downregulation on AcPP cell proliferation. **(A)** Proliferation cells (red fluorescence) in the AcPP of different treatment groups determined by EdU incorporation assay; Scale bar = 25 µm. **(B)** Percentage of EdU positive cells; it is to be noted that EdU positive cells were significantly fewer in the CALR-RNAi group than those in the other groups (***p < 0.001). **(C)** Proliferation of AcPP cells evaluated by CCK-8 assay on 6-day incubation. **(D)** Colony-forming units (CFUs) (top) and a single CFU (bottom) formed by different groups of AcPP cells on day 14 (scale bar = 500 µm). **(E)** Number of CFUs; it is to be noted that CFUs in the CALR-RNAi group were significantly fewer than those in the other groups. **p < 0.01 and ***p < 0.001.

Fewer proliferating cells (EdU positive) were detected in the CALR-RNAi group (15.00 ± 3.22%) than in the control (36.17 ± 5.19%) and vehicle (29.50 ± 3.72%) groups (*p* < 0.001; Figures 8A,B). Likewise, the proliferation rate of the AcPP cells in the CALR-RNAi group was significantly lower than that of the vehicle (*p* < 0.01) and control (*p* < 0.05) groups from day 3 onward after seeding (Figure 8C). Furthermore, the results of colony-forming unit (CFU) analysis showed that the CALR-RNAi group exhibited the lowest number of CFUs compared to those of the control (*p* < 0.01) and vehicle (*p* < 0.001) groups (Figures 8D,E). Taken together, the downregulation of CALR expression can effectively inhibit the proliferation of the AcPP cells *in vitro* and thus could be expected to suppress the initiation of antler regeneration *in vivo*.

### Downregulation of Calreticulin Expression Affected Cell Cycle Progression of Activated Pedicle Periosteum Cells

**FIGURE 9:**
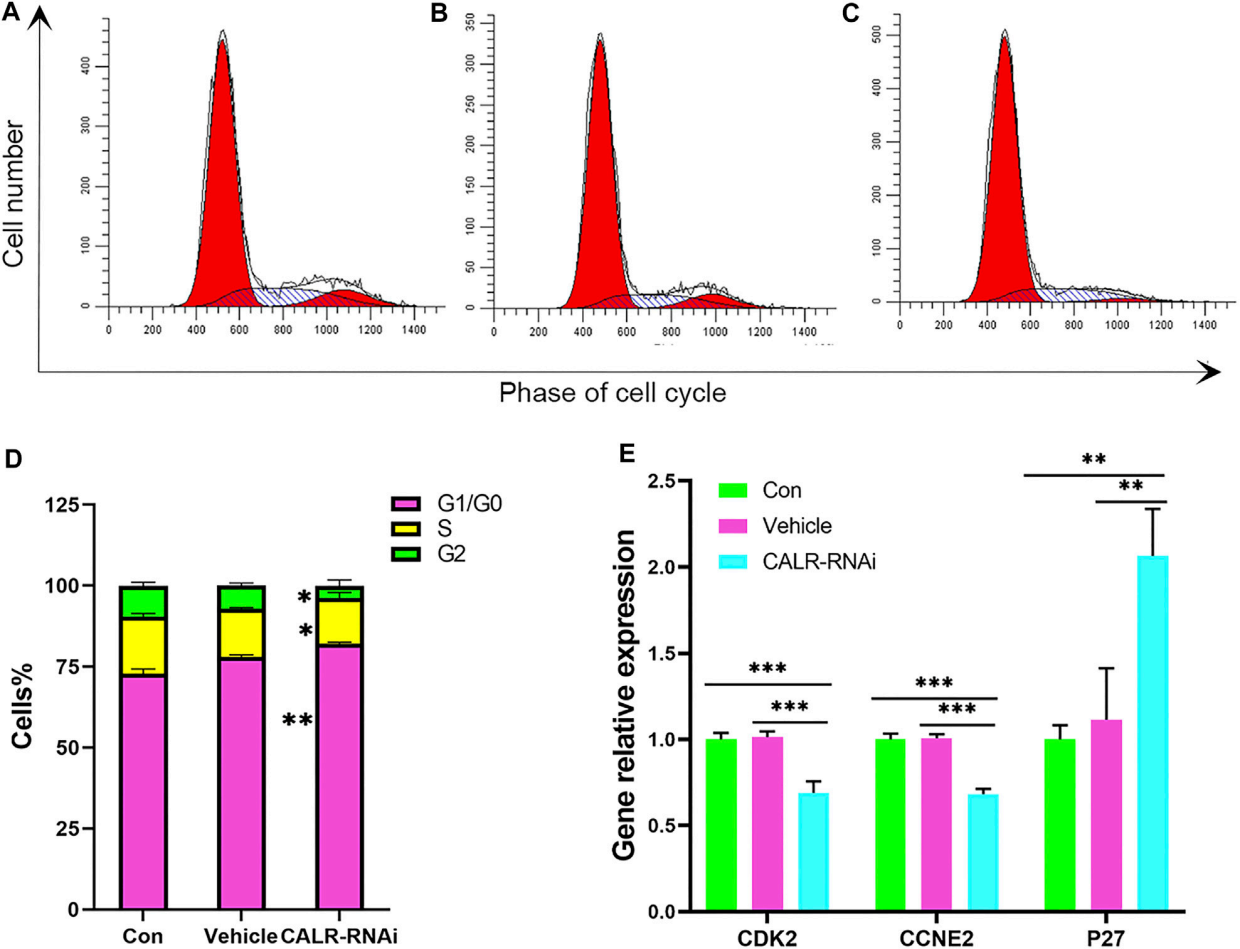
Effects of CALR downregulation on the AcPP cell cycle. **(A–C)** Cell cycle distribution of the AcPP cells after 24 h of culture. **(D)** Bar graphs depict the proportions of different phases (G1, S, and G2) accounting for each cell cycle after 24 h of culture; it is to be noted that CALR downregulation resulted in significant changes in the phase distribution. *p < 0.05 and **p < 0.01. **(E)** mRNA expression profiles of cell cycle regulatory genes, P27, CDK2, and CCNE2 in the control and CALR downregulated AcPP cells; it is to be noted that the cell cycle inhibitory gene (P27) was upregulated, whereas promoting genes (CDK2 and CCNE2) were downregulated. **p < 0.01 and ***p < 0.001.

To further assess the mitogenic effects of CALR on the AcPP cells, we measured the cell cycle progression using flow cytometry. The results showed that percentages of cells in the G0/G1 phase in the CALR-RNAi group (82.11 ± 0.37%) were significantly higher than those in the control (72.85 ± 1.46%) and vehicle (78.12 ± 0.52%) groups (*p* < 0.01). In contrast, cells in the S phase in the CALR-RNAi group (14.13 ± 0.95%) were significantly lower than those of the control group (17.63% ± 0.96%; *p* < 0.05) (Figures 9A–D). These data indicate that the downregulation of CALR in the AcPP cells can effectively induce cell cycle arrest by inhibiting G1-S phase transition.

The expression status of some key regulators of G1-S phase transition was then analyzed using qRT-PCR. The results showed that mRNA levels of cyclin E2 (CCNE2) and cyclin-dependent kinase 2 (CDK2) were decreased (*p* < 0.05), and the mRNA level of p27, a cell cycle inhibitor, was increased in the CALR-RNAi group compared to that in other groups (*p* < 0.05, Figure 9E).

### Downregulation of Calreticulin Expression Induced Apoptosis of the Activated Pedicle Periosteum Cells

**FIGURE 10:**
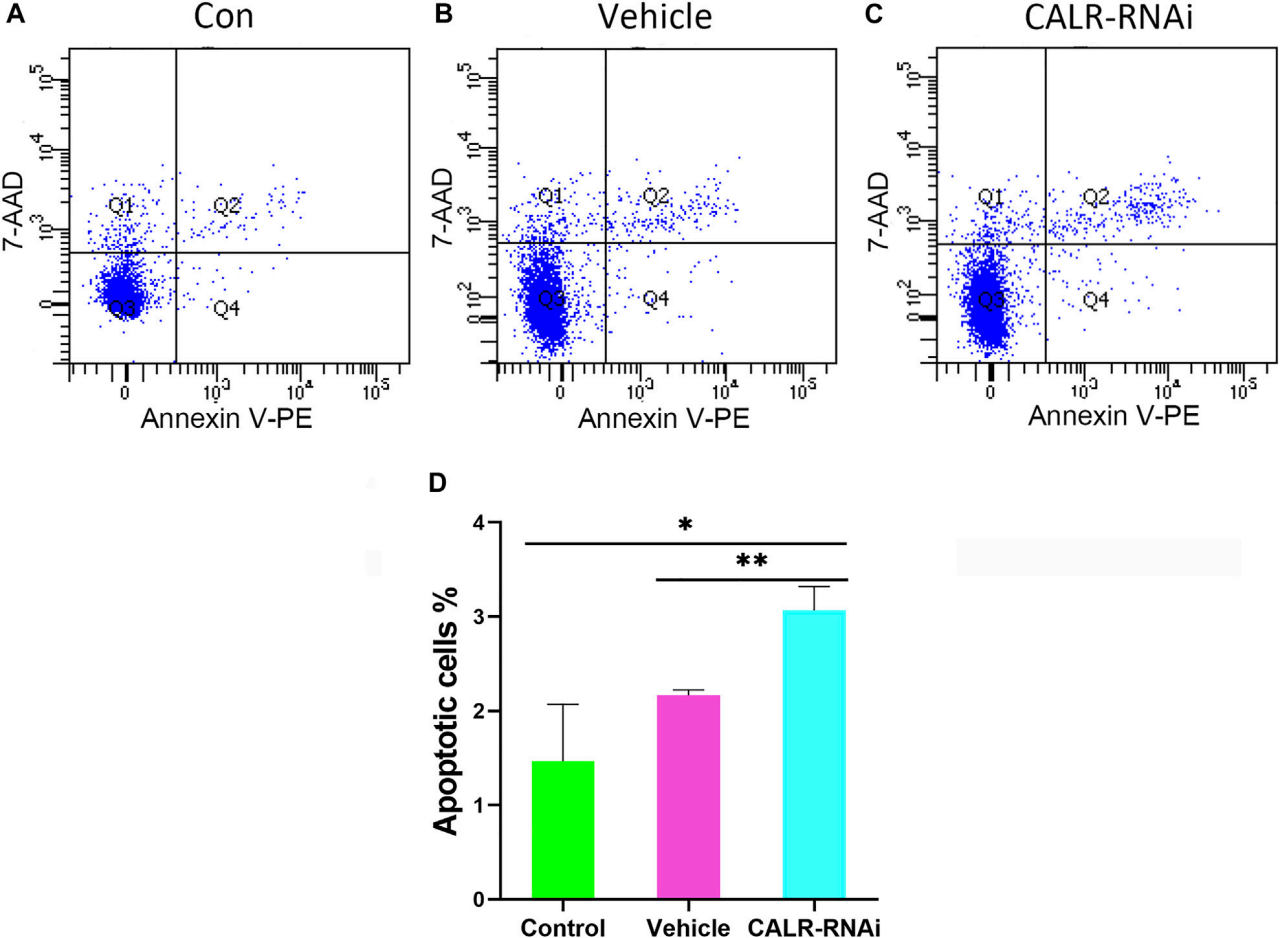
Effects of CALR downregulation on AcPP cell apoptosis. **(A–C)** Cell apoptosis analysis using flow cytometry. **(D)** Percentage of apoptotic cells in the different groups; it is to be noted that the apoptotic rate in the CALR-RNAi group was significantly increased compared to that in the other groups. *p < 0.05 and **p < 0.01.

Given the influence of CALR on proliferation of AcPP cells, we considered the effect of downregulation of CALR expression on apoptosis of these cells using flow cytometry. The results showed that the downregulation of CALR expression increased the apoptotic rate of the AcPP cells (*p* < 0.05, Figure 10), with the apoptotic rate of AcPP cells in the CALR-RNAi group (3.06 ± 0.25%) being higher than that of the control (1.46 ± 0.60%) and vehicle (2.16% ± 0.05%) groups (*p* < 0.05). These results suggest that the downregulation of CALR expression in the AcPP cells can also effectively induce AcPP cell apoptosis, besides inhibiting proliferation of these cells.

## Discussion

To the best of our knowledge, the present study is the first in the antler research field to investigate the transition process from the PoPP to the AcPP using RNA-seq. Notably, the AcPP cells, although being activated entering the proliferation state still, like their predecessors, the PoPP cells, retained some MSC characters, such as expression of MSC marker genes CD73, CD90, and CD105.

In total, 6,214 DEGs of the AcPP over the PoPP were detected including 1,548 upregulated and 4,666 downregulated. These DEGs were found to be mainly involved in the cell cycle pathway, protein procession in endoplasmic reticulum, calcium signaling pathway, osteogenesis, and immune response; all of these are consistent with our histological findings and functional analyses. Among these DEGs, CALR, an androgen response gene, was found to be highly significantly expressed in the cells of the AcPP over the PoPP. The expression level of CALR in these PP cells was causally related to the changes in the androgen hormone level *in vitro*. Taken together, these findings show that it is highly likely that CALR serves as one of the key regulators that drive the formation of antler blastema by activating/permitting AcPP cell proliferation.

Although it has been known for decades that antler regeneration can only take place when circulating androgen hormones decrease to the baseline (Akhtar et al., 2019), the factor(s) that are activated by the decrease in androgens and responsible for triggering initiation of antler regeneration have proven to be elusive. With the discovery of the PP as the tissue type that gives rise to regenerating antlers (Li et al., 2007) and the definition of the three differentiation states of the PP, namely, the DoPP, PoPP, and AcPP (Li et al., 2004; Li et al., 2005; Li, 2013), we are now ready to identify this molecule(s) using modern techniques.

In the present study, using RNA-seq, we found that CALR, an androgen response gene (Wang et al., 1997), was highly significantly expressed in the cells of the AcPP over the PoPP using both qRT-PCR and IHC. Subsequent experiments convincingly showed that the downregulation of CALR expression had significant impacts on the AcPP cells: impaired proliferation rate, inhibited G1-S phase transition in cell cycle progression, and induced apoptosis. Therefore, CALR is at least one of the androgen downstream mediators for triggering initiation of antler regeneration.

CALR is a highly conserved chaperone protein which resides primarily in the endoplasmic reticulum (Luo and Lee, 2013; Halperin et al., 2014) and is also located in the nucleus and cytoplasm, playing multiple roles in a variety of cellular processes (Coppolino et al., 1997; Krause and Michalak, 1997; Kielbik et al., 2021). CALR is reported to be involved in mediation of the effects of androgen hormones on cell proliferation and prevention of apoptosis in male sex accessory glands. For example, castration of rats downregulates CALR expression and then causes prostate involution (apoptosis), whereas androgen replacement upregulates CALR expression and then stimulates prostate regrowth (proliferation) back to the original size (Krause and Michalak, 1997; Zhu and Wang, 1999). In a recent report (Zheng et al., 2020), CALR knockdown in a cancer cell line NKTCL significantly reduced the cell proliferation rate, inhibited G1-to-S phase transition by increasing the expression level of p27, the cell cycle inhibitor, and decreasing the levels of cyclin E2 (CCNE2) and cyclin-dependent kinase 2 (CDK2). Antler stem cell activation (from the PoPP to the AcPP) and formation of regenerating antler blastema are essentially the reflection of cell proliferation and/or prevention of cell apoptosis. In the present study, we found that the downregulation of CALR expression in the AcPP cells (antler being another male accessory sex organ) *in vitro* significantly impaired the cell proliferation rate, arrested more cells at the G1 phase, increased expression level of p27, and decreased expression levels of CCNE2 and CDK2. Therefore, the observed decrease in the proliferation rate of AcPP cells by the downregulation of CALR expression may be achieved *via* inhibiting cell cycle progression at the phase of G1-to-S transition.

Zhu et al. (1998) reported that CALR downregulation causes cell apoptosis likely because a low level of CALR is insufficient to buffer the cytotoxic intracellular Ca^2+^ overload. As a high capacity intracellular Ca^2+^-binding protein, CALR alone binds approximately 50% of the Ca^2+^ stored in the endoplasmic reticulum lumen (Qiu et al., 2012; Venkatesan et al., 2021). The overexpression of CALR in a tumor cell line effectively increased the Ca^2+^ buffering capacity and protected cells against apoptosis (Bastianutto et al., 1995). In consistence with these findings, in the present study, we found that the CALR level in the AcPP cells was significantly upregulated when the level of circulating androgen decreased to the baseline, while the calcium signaling pathway was downregulated. Furthermore, the downregulation of CALR expression in the AcPP cells *via* RNAi *in vitro* increased cell apoptosis. Therefore, as a key regulator for Ca^2+^ homeostasis, we propose that a high level of CALR in the AcPP cells can reduce the cell stress caused by an overload of Ca^2+^ and effectively prevent cell apoptosis.

It has been well-established that androgen plays its role *via* its receptor (AR) and that the AR is a ligand-dependent transcription factor that regulates the expression of androgen response genes (Chang et al., 1995; Funahashi et al., 2015). Thus, these genes serve as the downstream mediators for the androgen effects. Among these genes, CALR was found to be positively regulated by androgens in the male sex accessory glands such as prostate and seminal vesicles but not in other organs such as the liver, brain, kidney, heart, and muscle (Wang et al., 1997). We have shown that CALR expression in the antler stem cells (AcPP cells) during regeneration of deer antlers was also regulated by androgens in the present study. However, unlike the other male sex accessory organs in which CALR expression is positively regulated by androgens, in the AcPP cells, it was negatively regulated; that is, a high level of CALR expression was associated with low levels of androgen. The phenomenon of androgen negatively regulating CALR has not been reported thus far in the classic sex accessory organs, but the results of the present study are supported by the findings from studies of our team: castration in the hard antler phase effectively induced hard antler casting and immediate regeneration of new antlers and at the same time triggered CALR expression in the distal PP tissue (equivalent to the AcPP; Akhtar et al., 2019); CALR was highly differentially expressed in the rapid proliferating cells in the antler growth center compared to the DoPP (dormant) cells (Dong et al., 2020).

Irrespective of negative or positive regulation by androgens, the CALR gene must be highly expressed in the activated PP cells and in the antler growth center cells as these proliferating cells require high levels of CALR for maintenance of Ca^2+^ homeostasis and prevention of apoptosis. Given that active cells demand increased protein synthesis (Feng et al., 2015), enhanced endoplasmic reticulum activity, such as the unfolded protein response (UPR), is required to facilitate protein folding and protect the cell from deleterious accumulation of unfolded proteins (Luo and Lee, 2013; Chen et al., 2022). As an endoplasmic reticulum resident protein, CALR is a well-established effector of the UPR (Schardt et al., 2011; Halperin et al., 2014), so the increased expression of CALR in activated PP cells may play a role in facilitating protein correct folding by mediating the UPR. Interestingly, recent reports have shown that androgens activate UPR signaling in both normal and cancer cells (Sheng et al., 2015; Jin and Saatcioglu, 2020). Therefore, the potential links between CALR, androgen, and UPR signaling during the process of PoPP activation would be of interest. In addition, the expression of CALR in the AP cells, another type of antler stem cell responsible for pedicle and first antler formation, is positively regulated by androgens during the initiation of pedicle formation (Liu et al., unpublished).

It is currently not known why and how regulation of CALR expression by androgens is altered in the antler regeneration. Zhu et al. (1998) reported that although CALR is an androgen response gene, the AR-binding site has not been identified from the CALR promoter region (Zhu et al., 1998). These authors put forward two explanations: 1) AR-binding sites may be localized far away from the transcription initiation site of the CALR promoter; 2) AR may regulate CALR gene expression without making direct contact with a DNA element(s) but rather by interacting with another DNA-binding protein.

If the second explanation (indirect regulation) applies, then the positive or negative effect of androgens on the regulation of CALR expression in the antler system would likely be dependent on the binding partners. Under this hypothesis, binding with partner X would result in positive regulation (such as with the prostate and AP cells), whereas binding with partner Y would result in negative regulation, such as in the PP cells. Analogous to this is the dual nature of regulation in angiogenesis. Thrombospondin-1 (TSP1) is a factor that can be pro-angiogenic or antiangiogenic solely depending on the binding partner with which the TSP1 interacts (Kaur et al., 2021): TSP1 inhibits angiogenesis if interacting with CD36, whereas TSP1 stimulates angiogenesis if it interacts with some integrin heterodimers (such as 3β1 and 6β1). If the partner-dependent theory is true, subsequent identification and characterization of these putative binding partners would be of great significance in developing an understanding of the mechanism underlying androgen regulation of CALR expression.

**FIGURE 11:**
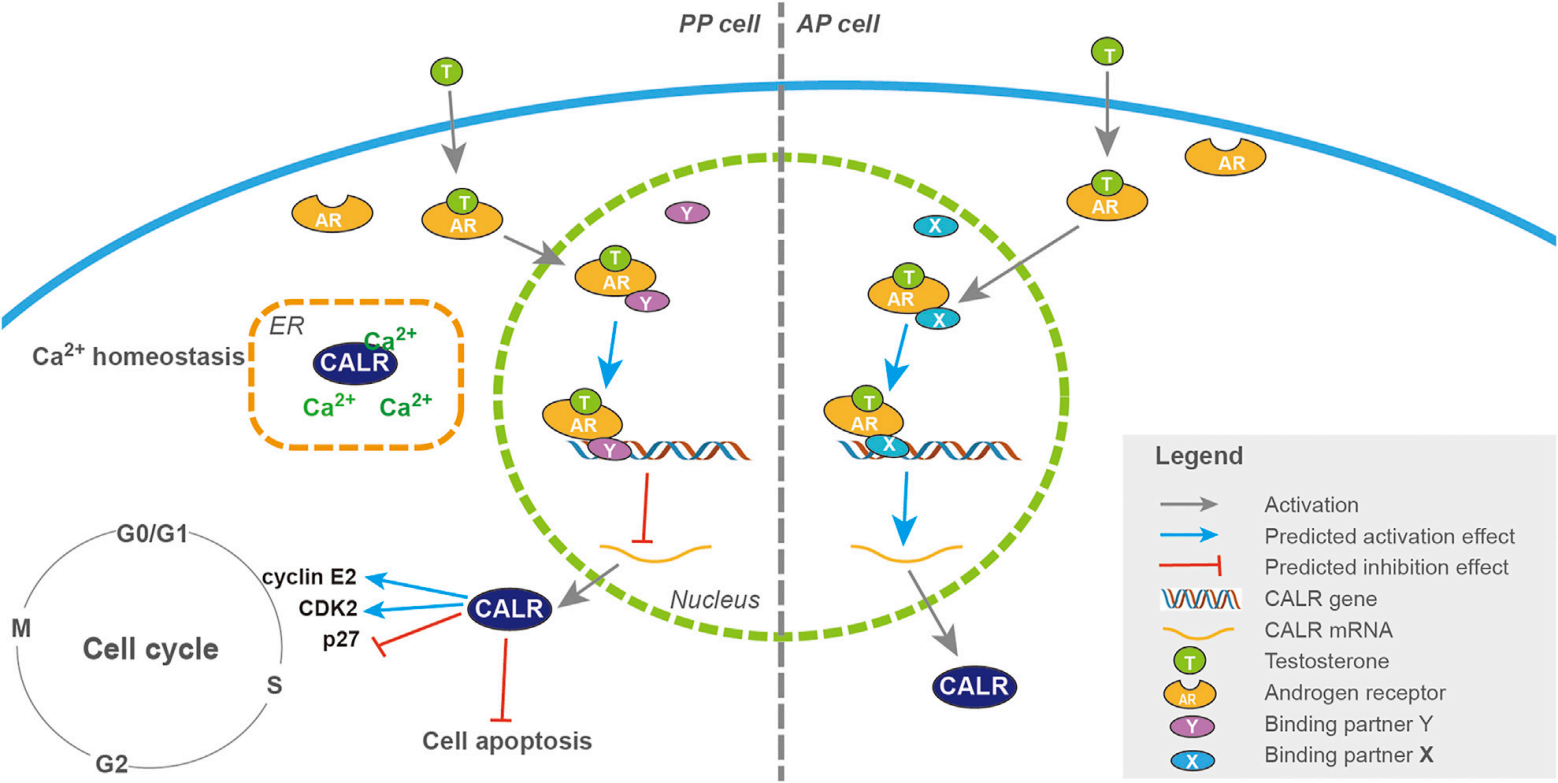
Pathways of CALR regulation by androgens in the activation of PP and AP cells during antler regeneration and pedicle formation, respectively. Schematic drawing of the regulatory pathways of CALR expression by androgen during initiation of antler regeneration (PP; left half) and pedicle formation (AP; right half). T binds to the AR once it has entered the cytoplasm, and the complex of AR + T interacts with a putative binding partner once translocated into the nucleus, and the triad (AR + T + partner) serves as a transcriptional factor either downregulating (with partner Y in the PP, refer to the left half) or upregulating (with partner X in the AP, refer to the right half) the expression of the CALR gene. A high level of CALR expression stimulates cell proliferation through facilitation of cell cycle progression (P27, CDK2, and cyclin E2).

With the discovery of the involvement of the androgen response gene, CALR, in the initiation of antler regeneration, we are now in the position to outline the whole picture of the initiation of antler regeneration (Figure 11). To help understand this regeneration process, we make an analogy with starting an automatic motor vehicle: PP at the DoPP stage is like a parked car with the brake on [gear in “P (parking)”]; potentiation of the PP (PoPP) through interactions with the enveloping skin is like turning on the car engine, but the car still remains unmoved as the gear is still in the position “P;” activation of the PP (AcPP) triggered by decreasing in the circulating androgen level to the baseline is like shifting the gear from “P” to “D (driving);” and commencement of AcPP cell proliferation under highly expressed CALR is like engagement of the motor power with the wheels so that the car is starting to move forward, provided that the fuel (high level of IGF1) is readily available in the tank.

Overall, we believe that identification of an androgen response gene, CALR, in the present study has not only discovered “one critical piece” of the “jigsaw puzzle” in the initiation of antler regeneration (helping to further reveal the mechanism underlying this unique mammalian epimorphic regeneration) but also opened up a new avenue for investigation of the nature of CALR regulation by androgen (putative binding partners). This latter aspect may be very relevant for seeking new targets for the treatment of androgen-related clinical conditions.

## Data Availability

The datasets presented in this study can be found in online repositories. The names of the repository/repositories and accession number(s) can be found below: NCBI BioSample accession number: SAMN17863449. The link is https://www.ncbi.nlm.nih.gov/biosample.
