# Supplementary figures and images for: Calreticulin Identified as One of the Androgen Response Genes That Trigger Full Regeneration of the Only Capable Mammalian Organ, the Deer Antler
Source: Front Cell Dev Biol. 2022 Jun 13;10:862841. doi: 10.3389/fcell.2022.862841 (PMC9235033; doi:10.3389/fcell.2022.862841)

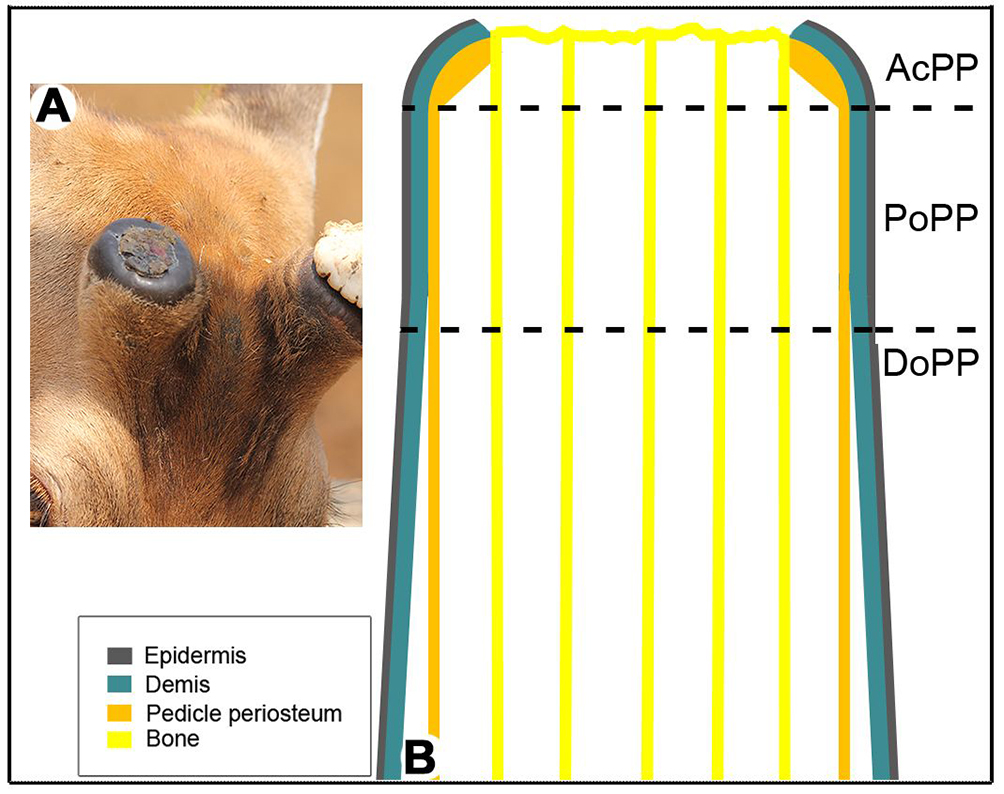

Supplement: Supplementary file 1 [file Image1.jpeg]
