# Supplementary material for: Calreticulin Identified as One of the Androgen Response Genes That Trigger Full Regeneration of the Only Capable Mammalian Organ, the Deer Antler
Source: Front Cell Dev Biol. 2022 Jun 13;10:862841. doi: 10.3389/fcell.2022.862841 (PMC9235033; doi:10.3389/fcell.2022.862841)
Supplement: Supplementary file 2 [file Table1.DOC]

**FIGURE S1.** Morphology and schematic diagram of a fresh pedicle stump to show location of different differentiation states of PP (orange)

**(A)** A pedicle stump immediately after hard antler casting. **(B)** Schematic drawing of the longitudinal cut surface of a pedicle stump immediately after hard antler casting. The three differentiation states of PP, AcPP, PoPP and DoPP, were delineated by the dotted lines. Note that DoPP is a thin layer that covers proximal two-thirds of the pedicle bone and loosely attached to the envelop skin; PoPP is a thin layer that covers a distal third of the pedicle bone and intimately associated to the envelop skin; AcPP is a thickened layer that encroaches peripherally onto the pedicle cast plane and intimately associated to the velvet antler skin.
